# Overfishing and climate change elevate extinction risk of endemic sharks and rays in the southwest Indian Ocean hotspot

**DOI:** 10.1371/journal.pone.0306813

**Published:** 2024-09-05

**Authors:** Riley A. Pollom, Jessica Cheok, Nathan Pacoureau, Katie S. Gledhill, Peter M. Kyne, David A. Ebert, Rima W. Jabado, Katelyn B. Herman, Rhett H. Bennett, Charlene da Silva, Stela Fernando, Baraka Kuguru, Robin W. Leslie, Meaghen E. McCord, Melita Samoilys, Henning Winker, Sean T. Fennessy, Caroline M. Pollock, Cassandra L. Rigby, Nicholas K. Dulvy

**Affiliations:** 1 Earth to Ocean Research Group, Department of Biological Sciences, Simon Fraser University, Burnaby, British Columbia, Canada; 2 Seattle Aquarium, Species Recovery Program, Seattle, Washington, United States of America; 3 Fish Ecology Lab, School of the Environment, University of Technology Sydney, Ultimo, New South Wales, Australia; 4 Molecular Breeding and Biodiversity Research Group, Stellenbosch University, Stellenbosch, Western Cape, South Africa; 5 South African Shark Conservancy, Hermanus, Western Cape, South Africa; 6 Research Institute for the Environment and Livelihoods, Charles Darwin University, Darwin, Northern Territory, Australia; 7 Pacific Shark Research Center, Moss Landing Marine Laboratories, Moss Landing, California, United States of America; 8 South African Institute for Aquatic Biodiversity, Makhanda, Eastern Cape, South Africa; 9 Elasmo Project, Dubai, United Arab Emirates; 10 Georgia Aquarium, Atlanta, Georgia, United States of America; 11 Wildlife Conservation Society, Makhanda, Eastern Cape, South Africa; 12 Department of Forestry, Fisheries and the Environment, Fisheries Research and Development Branch, Cape Town, Western Cape, South Africa; 13 Oceanographic Institution of Mozambique, Maputo, Mozambique; 14 Tanzania Fisheries Research Institute, Dar es Salaam, Tanzania; 15 CORDIO East Africa, Mombasa, Kenya; 16 British Columbia Chapter, Canadian Parks and Wilderness Society, Vancouver, British Columbia, Canada; 17 Oceanographic Research Institute, Durban, KwaZulu-Natal, South Africa; 18 IUCN Red List Unit, Cambridge, United Kingdom; 19 College of Science and Engineering, James Cook University, Townsville, Queensland, Australia; COISPA Tecnologia & Ricerca - Stazione Sperimentale per lo Studio delle Risorse del Mare, ITALY

## Abstract

Here, we summarise the extinction risk of the sharks and rays endemic to coastal, shelf, and slope waters of the southwest Indian Ocean and adjacent waters (SWIO+, Namibia to Kenya, including SWIO islands). This region is a hotspot of endemic and evolutionarily distinct sharks and rays. Nearly one-fifth (*n* = 13 of 70, 18.6%) of endemic sharks and rays are threatened, of these: one is Critically Endangered, five are Endangered, and seven are Vulnerable. A further seven (10.0%) are Near Threatened, 33 (47.1%) are Least Concern, and 17 (24.3%) are Data Deficient. While the primary threat is overfishing, there are the first signs that climate change is contributing to elevated extinction risk through habitat reduction and inshore distributional shifts. By backcasting their status, few endemic species were threatened in 1980, but this changed soon after the emergence of targeted shark and ray fisheries. South Africa has the highest national conservation responsibility, followed by Mozambique and Madagascar. Yet, while fisheries management and enforcement have improved in South Africa over recent decades, substantial improvements are urgently needed elsewhere. To avoid extinction and ensure robust populations of the region’s endemic sharks and rays and maintain ecosystem functionality, there is an urgent need for the strict protection of Critically Endangered and Endangered species and sustainable management of Vulnerable, Near Threatened, and Least Concern species, underpinned by species-level data collection and reduction of incidental catch.

## Introduction

Anthropogenic pressures are mounting in the global oceans, and extinction risk is increasing mainly due to overfishing [1–4]. Over 1,500 marine species are threatened globally; more than two-thirds of these are threatened due to overfishing, more than double the risk caused by the next most-cited threat (residential and commercial development) [5]. The international community has formally agreed through the Convention on Biological Diversity that extinctions be prevented, yet 2020 marine targets were not met, namely Aichi Targets 6 (fisheries sustainability), 11 (avoiding extinction risk), and 14 (life under water). The international community agreed in late 2022 to halt extinction of known threatened species now and recover native species to healthy and resilient levels by 2050 under the new Kunming-Montreal Global Biodiversity Framework (Goal A, Targets 4 and 5) [6]. The IUCN Red List of Threatened Species provides a robust assessment of extinction risk, especially when tracked over time through the Red List Index [7–9]. Therefore, there is a need to expand the Red List Index particularly for marine fishes threatened by overfishing.

Marine taxa particularly threatened by fisheries include the sharks and rays (subclass Elasmobranchii), which are directly targeted or captured incidentally in fisheries targeting more productive species [4]. Sharks and rays represent an ancient lineage of over 400 million years of evolution [10]. Further, they often function as apex and mesopredators in pelagic, benthic, and nearshore environments [11]. Understanding how changes in fisheries management are affecting sharks and rays through the Red List Index is crucial to gauging progress toward international biodiversity targets. The first comprehensive assessment of this unique radiation of fishes, published in 2014, estimated that over one-quarter are threatened [12]; the first reassessment completed in 2021 reveals that over one-third are now threatened [4]. Some progress has been made thus far with taxonomic and regional species subsets to track change through reassessment and Red List Index development [9, 13–16].

The southwest Indian Ocean (SWIO) and adjacent waters (from Namibia to Kenya, including the Benguela current, waters off East Africa, and SWIO islands, hereafter SWIO+) have among the most distinctive shark and ray faunas globally, comprised of high richness and endemicity with many evolutionarily distinct species [17, 18]. This area harbours over 250 species from at least 47 families, in part due to the diversity of habitats, from coral and rocky reefs to mangroves, kelp and soft sediment habitats, within tropical, sub-tropical, warm-temperate, and cool-temperate biogeographical regions [19, 20]. The biogeography is influenced in the south by the unique ecological conditions created by the confluence of the warm southward-flowing Agulhas Current along the east and south coasts of South Africa and the cold northward-flowing Benguela Current on the west coast of South Africa and Namibia [20].

Coastal regions of SWIO+ are under considerable fishing pressure. Approximately one-quarter of the human population lives within 100 km of the coast and population growth is among the highest worldwide, with a projected doubling of the human population by 2050 [21]. Coastal communities in the region are heavily dependent on fisheries as the primary source of protein, livelihoods, and food security [22, 23]. The pressure and scale of artisanal fisheries are significant and could pose an equivalent if not greater threat than industrialized fleets to sharks in the region. For example, in Mozambique, the total small-scale fisheries catch is estimated to be as much as three times that of the industrial sector [24]. Several nations in the SWIO+ region face significant socio-economic challenges and rank in the lowest quartile of the Human Development Index (HDI) [25], limiting their ability to manage marine resources effectively. This includes sharks and rays, which are subject to generally unregulated take in parts of SWIO+, particularly in artisanal fisheries [26–28].

Here, we provide an assessment of extinction risk status of 70 shark and ray species endemic to SWIO+. Specifically, we: (1) assess the extinction risk of these sharks and rays using the IUCN Red List Categories and Criteria, (2) compare the change in extinction risk over ~40 years against a retrospective assessment for 1980 using the Red List Index, and (3) determine the countries with the most significant national conservation responsibility with respect to sharks and rays. Finally, we propose some general policies that, if implemented, will help to safeguard shark and ray populations in SWIO+.

## Methods

We first describe the geographic and taxonomic scope of the regional endemic shark and ray extinction risk assessment, followed by the application of the IUCN Red List Categories and Criteria, species mapping and spatial analyses, and the calculation of a Red List Index.

### Geographic and taxonomic scope

We focus on the assessment of extinction risk in endemic sharks and rays of the SWIO+ that inhabit the continental and insular shelves and slopes off Africa from the Angola–Namibia border, around the Cape of Good Hope, and east and north to the Kenya–Somalia border. The region also includes Madagascar and the islands of the southwest Indian Ocean. The geographic scope thus comprised nine range countries: Namibia, South Africa, Mozambique, Tanzania, Kenya, Madagascar, Comoros, Seychelles, and Mauritius. The Namibia–Angola border was chosen as the northwestern boundary of the region because of the oceanographic and faunal break at the interface between the Benguela and Guinea Currents [20]. The Kenya–Somalia border was chosen as the northeastern-most limit of this assessment as it abuts the boundary of the Arabian Sea and its adjacent waters region, the subject of a separate recent Red List assessment [29]. The French overseas departments of Réunion and Mayotte are not included here because no regionally endemic sharks or rays are known to exist there. We caution that with recent discoveries of guitarfishes, sawsharks, and numerous deepwater catsharks, that parts of this region remain poorly surveyed and new surprises await us [30–32]. We collectively refer to the region studied here as SWIO+ for accuracy and simplicity.

A comprehensive list of all shark and ray species known to occur in the region provided the taxonomic foundation for our assessment [33, 34]. We evaluated 70 shark and ray species considered endemic to the region and did not include those that inhabit wider-ranging coastal, pelagic, or deepwater areas. For nomenclature and taxonomy, we followed the online electronic version of the Catalog of Fishes for sharks [34] and *Rays of the World* for rays [35, 36].

### Application of the IUCN Red List Categories and Criteria

We assessed species at the global level by applying the IUCN Red List Categories and Criteria (Version 3.1) and the associated guidelines [37, 38]. Existing data and information on each species, including taxonomy, geographic distribution, population trends, habitat and ecology, significant threats, and conservation measures were compiled by the IUCN Species Survival Commission Shark Specialist Group (hereafter ‘SSG’) and regional experts. Information was obtained from published peer-reviewed scientific literature, government reports, unpublished fisheries data, grey literature, and expert personal observations and unpublished data.

A four-day workshop was convened at the National Research Foundation’s South African Institute for Aquatic Biodiversity (SAIAB) in Grahamstown in April 2018, facilitated by the SSG. Workshop participants included regional fisheries, biodiversity, and taxon-specific experts, including representatives of non-governmental organizations, fisheries agencies, and government staff from countries across the SWIO+ region. During the workshop, participants shared data, reports, and expertise for each species and threats from the region. This group systematically assessed these 70 species against each of five quantitative IUCN Red List Criteria A–E: A, population reduction; B, geographic range; C, small population size and decline; D, very small or restricted population; and, E, quantitative analysis [37].

Each species was assigned to one of the following Red List Categories: Extinct (EX), Extinct in the Wild (EW), Critically Endangered (CR), Endangered (EN), Vulnerable (VU), Near Threatened (NT), Least Concern (LC), or Data Deficient (DD) (for definitions, see [37]. The categories CR, EN, and VU are collectively termed ‘threatened’ categories. A species qualifies for one of the three threatened categories by meeting the quantitative threshold for that category within one of the five Criteria (A–E). The NT category is applied to species that approach, but do not meet, a threshold for a threatened category. The LC category is applied to species that have been assessed against the Red List Criteria but do not qualify for CR, EN, VU, or NT. There were two ways species were assessed as LC: (i) data show that the species has a stable or increasing population size over three generation lengths (3GL), or (ii) the species inhabits remote or deepwater areas that are not subject to known threats and therefore it can be inferred that the population is not undergoing reduction. The DD category is applied to a species when there is inadequate information to make a direct or indirect assessment of the risk of extinction based on its distribution and/or population status [38]. The Red List assessment process includes a structured approach to classifying threats into 11 primary classes, such as *Residential & commercial development*, *Biological resource use*, and *climate change & severe weather* [39] and appropriate threats were selected for each species. *Biological resource use* encompasses “threats from consumptive use of ‘wild’ biological resources including deliberate and unintentional harvesting effects”. Specifically, species were classified under the secondary code 5.4 *Fishing & harvesting aquatic resources* if they were suspected, inferred, or observed to be captured in fisheries based on the workshop process and subsequent review of literature.

Red List Criterion A uses a set of quantitative thresholds to classify population reduction over the past 3GL [37]. One primary source of long-term abundance data for 17 species analysed here is demersal research trawl surveys conducted in South Africa during summer along the west coast and autumn and spring along the south coast by the Fisheries Research and Development Branch of the Department of Forestry, Fisheries, and the Environment (DFFE) (DFFE unpubl. data 2018). Annual density estimates (kg per nm^2^ area swept) were estimated using the geostatistical delta-generalized linear mixed model (GLMM) developed by Thorson *et al*. [40]. Applications of the delta-GLMM to South African trawl survey index standardization have been described elsewhere [41, 42] and the spatial patterns in density over time are shown for species for which there were data available. Although demersal trawl surveys commenced in 1984, we only considered the period from 1991 onwards due to improvements in species identification following the initial survey years. The second source of data was angling records (number of fish per angler per day) provided from the De Hoop Marine Protected Area (MPA) shore angling surveys conducted jointly by the South African Department of Environmental Affairs (now a part of the Department of Forestry, Fisheries, and the Environment (DFFE), unpubl. data 2018) and the Department of Biological Sciences, Marine Research Institute, University of Cape Town. The angler-standardized catch-per-unit-effort (CPUE) data (1997–2017) provided were already standardized by routine methods from the government (GLMM standardization for season, fishing techniques, year, and stratified location). All datasets underwent extensive checks before analyses, and their reliability was reviewed by experts during the workshop. For the analysis, each survey season was treated as an individual index *i*.

To analyze trend data, we used a Bayesian population state-space model designed specifically for IUCN Red List assessments (‘Just Another Red List Assessment’, JARA) [9, 43, 44], which builds on the Bayesian state-space tool for averaging relative abundance indices [45] and is available in an R package on the GitHub open-source repository (www.github.com/henning-winker/JARA; JARA v.1.1.1). Each relative abundance index (or time-series) was assumed to follow an exponential growth process defined through the state process equation:

$$\mu_{t+1}=\mu_{t}+r_{t}$$

where *μ*_*t*_ is the logarithm of the expected abundance in year *t*, and *r*_*t*_ is the normally distributed annual rate of change with mean $\bar{r}$, the estimable mean rate of change for a time-series, and process variance *σ*^2^. We linked the logarithm of the observed relative abundance indices to the logarithm of the true expected population trend using the observation equation (eqn. 16 from Winker *et al*. [45]). We used a non-informative normal prior for $\bar{r}∼N(0,1000)$, and an approximately uniform prior on the log scale for the process variance $\sigma^{2}∼\frac{1}{gamma(0.001,0.001)}$.

We ran three Monte Carlo Markov chains for each dataset with different initial values. Each Markov chain was initiated by assuming a prior distribution on the initial condition centred around the first data point in each abundance time-series. In each chain, the first 30,000 iterations were discarded (‘burn-in’), and of the remaining 60,000 iterations, 10,000 were selected for posterior inference (‘thinning rate’ = 6) from each chain. Thus, posterior distributions were estimated from 30,000 iterations. Convergence was diagnosed using Geweke’s diagnostic [46] with thresholds of p = 0.05 via the ‘coda’ library (v0.19–1) [47]. We conducted posterior predictive checks (drawing simulated values from the joint posterior predictive distribution of replicated data and comparing these samples to the observed data) by checking that the credible interval of the fit of the models fall each time within the posterior predictive distribution limits [48]. The Highest Posterior Density interval was used as the interval estimator of 95% credible intervals. Analyses were performed using R Statistical Software v3.5.0 [49] and via the package JARA v1.1.1 [44].

While there are many demographic approaches to calculating generation length [24], these are generally data-intensive and have been applied to relatively few sharks and rays. Therefore, to derive GL, a simple measure that requires only female age-at-maturity and maximum age was used:

GL=maximumage+(maximumage–age‐at‐maturity)*z,

where z depends on the mortality rate of adults and is typically around 0.3 for mammals but we assume z is 0.5 to account for the truncation of age-structure due to overfishing and underestimation of age in chondrichthyans [15, 43]. This value represents the median age of parents of the current cohort.

If a species qualified for a change in Red List Category from a previously published assessment, changes were classified as either *genuine* or *non-genuine* changes. Genuine changes are assigned due to actual increases or decreases in the level of extinction risk that a species faces based on changes in threatening processes. In contrast, non-genuine changes are assigned due to new information, taxonomic changes, and/or errors in the application of Criteria or incorrect data used in the previous assessment [4, 38].

Assessments were drafted after the workshop’s conclusion and the Category and Criteria, and assessment rationale sections were initially sent to all workshop participants to solicit feedback before circulation to the full membership of the SSG comprising 177 members from 55 countries for their input. Each assessment was peer-reviewed by at least two experts with knowledge of the species and the IUCN Red List Categories and Criteria. Completed assessments were submitted to the IUCN Red List Unit in Cambridge, UK, for final review and accepted for publication on the IUCN Red List.

### Attitude to risk and classification of uncertainty

In addition to the use of the JARA decision support tool to minimize conflict over the choice of data and model structures, the application of the IUCN Red List Categories and Criteria was improved over the previous assessment for three further reasons: (i) application of a precautionary mindset to Red List assessments, (ii) better understanding of the alignment of fisheries stock assessments and IUCN Red List Criteria, and (iii) avoidance of consideration of ‘downstream consequences’ in status assessment [4, 43].

First, the IUCN guidelines state that global assessments should adopt a precautionary but realistic attitude and resist an evidentiary attitude, see section 3.2.3, p. 23 of the IUCN Red List Guidelines [38]. The guidelines caution that the assessor’s risk tolerance used to evaluate information can fall along an axis of evidentiary (high risk tolerance) to precautionary (low risk tolerance), where an evidentiary attitude will classify a species as threatened only when there is strong evidence (i.e., quantitative monitoring data) to support a threatened classification. Here, we use overlap of known or suspected fishing pressure with the depth and geographic range of each species, combined with life history traits, to arrive at a precautionary but realistic assessment of extinction risk.

Second, the previously evidentiary attitude arose from early concerns over the applicability of extinction risk Criteria, to wide-ranging exploited marine fishes: these early concerns have not been borne out [50, 51]. Since then, a large body of simulation and meta-analysis has demonstrated strong alignment between the fisheries status of species and the Red List status, including for chondrichthyans [52, 53].

Third, IUCN guidelines recommend assessors avoid ‘downstream’ consequences of a listing in decision-making. Three examples include: (1) a species being listed in one of the threatened categories, which might lead to strict protection curbing fisheries operations, (2) the listing of a species as DD might incentivise greater research funding, or (3) downlisting from threat category to NT or LC might lead to the removal of prohibition on retention of the species (see section 3.2.3, p. 23 of the IUCN Red List Guidelines [26]).

#### Species distribution mapping

Draft species range maps were primarily based on the original maps published in previous Red List assessments [12] augmented by revised distributions from those in *Sharks of the World* [54] and *Rays of the World* [36]. Maps were reviewed and validated by regional experts and taxonomists and the final distribution maps were prepared using ArcGIS 10.6. The ranges of each species were clipped to their known depth range based on the highest-resolution bathymetry dataset available across the region (15 arc seconds) [55]. One species, Kaja’s Sixgill Sawshark (*Pliotrema kajae*), was excluded from all spatial analyses, as it was not possible to map its range due to a lack of data [56].

#### Red List Index

We derived retrospective assessments for two earlier periods, 2005 and 1980 (with the current assessments set at 2020), to calculate a Red List Index (RLI) [9, 13–16]. Before this current reassessment, all except 15 newly described species had assessments published on the IUCN Red List. All changes in Red List category except one were considered non-genuine changes due to new information [38]. In other words, if what is currently understood was known during the previous assessments, the assigned status of those species would likely have been different. For example, if a species was assessed as DD in 2005 but is now LC in the current assessment, the older status would be retrospectively corrected to be LC. For species assessed as NT or in one of the threatened categories, backcasting was undertaken by retrospectively assigning status based on current understanding of the spatial and temporal pattern of coastal human population growth, the development of general fishing pressure, the availability of fishing gear capable of capturing sharks and rays, and the development of the international trade demand for shark and shark-like ray fins [9, 13–16].

The RLI for all 70 endemic species was also disaggregated to each of the nine SWIO+ range countries. The disaggregation of RLI to country level, which considers the relative proportions of all species’ ranges occurring in each country, allows a more nuanced understanding of which range countries contribute most to the change in Red List statuses across all species and the region. This is an important consideration because different range countries can potentially contain highly differing proportions of an individual species’ geographic range, driving or preventing extinction risk in this species. For calculating country specific RLI values, the equation is amended such that:

$${RLI}_{\left(t,u\right)}=1-\left[\frac{\sum \left(W{}_{\left(t,s\right)}\times \frac{r_{su}}{R_{s}}\right)}{WEX\times \sum \left(\frac{r_{su}}{R_{s}}\right)}\right]$$

where *t* is the year of assessment, *u* is the country and *W*_(*t*,*s*)_ is the Red List Category at year *t* for each species, multiplied by $\frac{r_{su}}{R_{s}}$, representing the proportion of each species’ total range found within the Exclusive Economic Zone (EEZ) of each country [57]. The threat score *W*_(*t*,*s*)_ in each year is the Red List Category in each year weighted according to risk with highest weighting of 4 for CR, 3 for EN, 2 for VU, 1 for NT) This is summed across all species that occur in each country’s EEZ and divided by the maximum threat score (*WEX* = 5), multiplied by the sum of proportional species’ ranges. The final country specific RLI value is derived by subtracting from 1. Higher RLI values indicate fewer negative changes in Red List status across species and vice versa (as with the global RLI). Finally, we calculated national conservation responsibilities for all range countries, which are based on the sum of all threat scores across species within a country, multiplied by each of the species’ proportional ranges for that country [58].

## Results

### Taxonomic diversity and species richness

**Fig 1:**
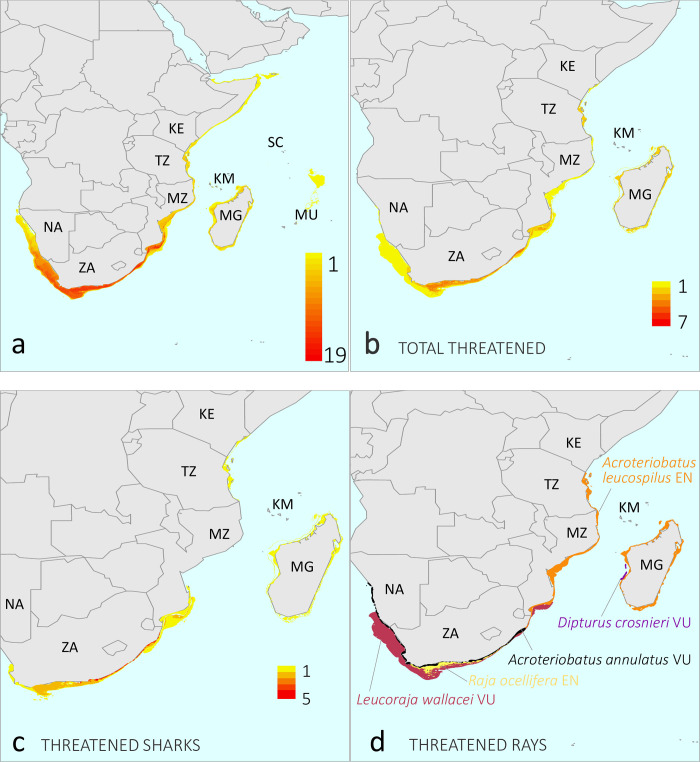
Endemic species richness of (a) sharks and rays (*n* = 70), (b) threatened (Critically Endangered, Endangered or Vulnerable, according to the IUCN Red List Categories) sharks and rays (*n* = 13), (c) threatened sharks (*n* = 8), and (d) individual distributions of threatened rays (*n* = 5) across the SWIO+ region. Maps made with Natural Earth.

This study area includes 70 endemic species (38 sharks and 32 rays, the latter comprising guitarfishes, electric rays, and skates) from 7 orders, 20 families, and 39 genera (Table 1). Endemic species richness is greatest along the South African and southern Mozambican coastlines, with a maximum number of 19 species occurring in each country (Fig 1A). The richness of threatened (Critically Endangered, CR; Endangered, EN; or Vulnerable, VU) shark and ray species reflects this inverse latitudinal gradient (*n* = 13; Fig 1B). The high concentration of threatened endemics in South Africa is driven by the sharks (*n* = 8), whereas threatened rays (n = 5) were more disparately distributed across the region (Fig 1C and 1D). Families with the highest species richness were Rajidae (hardnose skates, *n* = 12, 17.1% of all species) and Pentanchidae (deepwater catsharks, *n* = 15, 21.4%), collectively comprising more than a third (38.5%) of the regional endemic fauna.

**Table 1:** Original, backcast (for years 1980 and 2005), and current assessments of IUCN Red List categories for all endemic shark and ray species of the SWIO+ region (n = 70). Differences in original past assessments and backcast assessments arise due to new information about a species’ status from the better informed, more recent assessments.

Order	Family	Latin binomial	Common Name	Original IUCN Red List status			Red List status for RLI		
				2000	Mid-2000s	Late-2010s	1980	2005	2020
Squaliformes	Squalidae	Squalus acutipinnis*	Bluntnose Spurdog			NT_2019_	LC	LC	NT
Squaliformes	Squalidae	Squalus bassi*	African Longnose Spurdog			LC_2019_	LC	LC	LC
Squaliformes	Squalidae	*Squalus lalannei*	Seychelles Spurdog		DD_2008_	LC_2018_	LC	LC	LC
Squaliformes	Centrophoridae	*Centrophorus seychellorum*	Seychelles Gulper Shark		DD_2008_	LC_2018_	LC	LC	LC
Squaliformes	Etmopteridae	Etmopterus compagnoi*	Brown Lanternshark			LC_2018_	LC	LC	LC
Squaliformes	Etmopteridae	Etmopterus sculptus*	Sculpted Lanternshark			LC_2018_	LC	LC	LC
Squaliformes	Etmopteridae	Etmopterus sentosus*	Thorny Lanternshark		LC_2006_	LC_2018_	LC	LC	LC
Pristiophoriformes	Pristiophoridae	Pliotrema annae*	Anna’s Sixgill Sawshark			DD_2020_	DD	DD	DD
Pristiophoriformes	Pristiophoridae	Pliotrema kajae*	Kaja’s Sixgill Sawshark			DD_2020_	DD	DD	DD
Pristiophoriformes	Pristiophoridae	Pliotrema warreni*	Warren’s Sixgill Sawshark			LC_2019_	LC	LC	LC
Squatiniformes	Squatinidae	*Squatina africana*	African Angelshark		DD_2004_	NT_2017_	LC	LC	NT
Orectolobiformes	Hemicylinder	Chiloscyllium caeruleopunctatum*	Bluespotted Bambooshark			DD_2019_	DD	DD	DD
Orectolobiformes	Ginglymostomatidae	*Pseudoginglymostoma brevicaudatum*	Shorttail Nurse Shark		VU_2004_	CR_2018_	LC	VU	CR
Carcharhiniformes	Pentanchidae	*Apristurus saldanha*	Saldanha Catshark		LC_2004_	LC_2018_	LC	LC	LC
Carcharhiniformes	Pentanchidae	*Bythaelurus clevai*	Broadhead Catshark		DD_2004_	DD_2018_	DD	DD	DD
Carcharhiniformes	Pentanchidae	Bythaelurus lutarius*	Mud Catshark			DD_2018_	DD	DD	DD
Carcharhiniformes	Pentanchidae	Bythaelurus tenuicephalus*	Narrowhead Catshark			LC_2018_	LC	LC	LC
Carcharhiniformes	Pentanchidae	*Halaelurus lineatus*	Lined Catshark		DD_2004_	LC_2018_	LC	LC	LC
Carcharhiniformes	Pentanchidae	*Halaelurus natalensis*	Tiger Catshark		DD_2004_	VU_2018_	LC	NT	VU
Carcharhiniformes	Pentanchidae	*Haploblepharus edwardsii*	Happy Eddie Catshark	NT	NT_2008_	EN_2019_	LC	NT	EN
Carcharhiniformes	Pentanchidae	*Haploblepharus fuscus*	Brown Shyshark	NT	VU_2008_	VU_2019_	LC	VU	VU
Carcharhiniformes	Pentanchidae	*Haploblepharus kistnasamyi*	Natal Shyshark		CR_2008_	VU_2019_	LC	NT	VU
Carcharhiniformes	Pentanchidae	*Haploblepharus pictus*	Dark Shyshark		LC_2008_	LC_2018_	LC	LC	LC
Carcharhiniformes	Pentanchidae	*Holohalaelurus favus*	Honeycomb Izak Catshark		EN_2008_	EN_2019_	LC	EN	EN
Carcharhiniformes	Pentanchidae	*Holohalaelurus grennian*	Grinning Izak Catshark		DD_2008_	DD_2019_	DD	DD	DD
Carcharhiniformes	Pentanchidae	*Holohalaelurus melanostigma*	Crying Izak Catshark		DD_2006_	LC_2019_	LC	LC	LC
Carcharhiniformes	Pentanchidae	*Holohalaelurus punctatus*	African Spotted Catshark		EN_2008_	EN_2019_	LC	EN	EN
Carcharhiniformes	Pentanchidae	*Holohalaelurus regani*	Izak Catshark		LC_2007_	LC_2019_	LC	LC	LC
Carcharhiniformes	Scyliorhinidae	*Cephaloscyllium sufflans*	Balloon Shark		LC_2004_	NT_2019_	LC	LC	NT
Carcharhiniformes	Scyliorhinidae	*Poroderma africanum*	Pyjama Shark	NT	NT_2005_	LC_2019_	LC	LC	LC
Carcharhiniformes	Scyliorhinidae	*Poroderma pantherinum*	Leopard Catshark		DD_2004_	LC_2019_	LC	LC	LC
Carcharhiniformes	Scyliorhinidae	*Scyliorhinus capensis*	Yellowspotted Catshark	NT	NT_2004_	NT_2019_	LC	LC	NT
Carcharhiniformes	Scyliorhinidae	*Scyliorhinus comoroensis*	Comoro Catshark		DD_2007_	DD_2018_	DD	DD	DD
Carcharhiniformes	Proscyllidae	*Eridacnis sinuans*	African Ribbontail Catshark		LC_2004_	LC_2018_	LC	LC	LC
Carcharhiniformes	Triakidae	*Mustelus palumbes*	Whitespotted Smoothhound		DD_2006_	LC_2019_	LC	LC	LC
Carcharhiniformes	Triakidae	*Scylliogaleus quecketti*	Flapnose Houndshark	VU	VU_2005_	VU_2018_	LC	NT	VU
Carcharhiniformes	Triakidae	*Triakis megalopterus*	Spotted Gully Shark	NT	NT_2005_	LC_2019_	LC	LC	LC
Torpediniformes	Narcinidae	*Narcine insolita*	Madagascar Numbfish		DD_2004_	DD_2018_	DD	DD	DD
Torpediniformes	Narkidae	*Electrolux addisoni*	Ornate Sleeper Ray		CR_2008_	LC_2018_	LC	LC	LC
Torpediniformes	Narkidae	*Heteronarce garmani*	Natal Sleeper Ray		VU_2007_	NT_2019_	NT	NT	NT
Torpediniformes	Narkidae	*Narke capensis*	Cape Sleeper Ray		DD_2007_	LC_2018_	LC	LC	LC
Torpediniformes	Torpedinidae	Tetronarce cowleyi*	South African Torpedo			LC_2018_	LC	LC	LC
Torpediniformes	Torpedinidae	*Torpedo fuscomaculata*	Blackspotted Torpedo		DD_2004_	DD_2018_	DD	DD	DD
Rhinopristiformes	Rhinobatidae	*Acroteriobatus annulatus*	Lesser Guitarfish		LC_2006_	VU_2019_	LC	NT	VU
Rhinopristiformes	Rhinobatidae	*Acroteriobatus blochii*	Bluntnose Guitarfish		LC_2006_	LC_2018_	LC	LC	LC
Rhinopristiformes	Rhinobatidae	*Acroteriobatus leucospilus*	Greyspot Guitarfish		DD_2008_	EN_2018_	LC	VU	EN
Rhinopristiformes	Rhinobatidae	*Acroteriobatus ocellatus*	Speckled Guitarfish		DD_2008_	DD_2018_	DD	DD	DD
Rhinopristiformes	Rhinobatidae	*Rhinobatos austini*	Austin’s Guitarfish			DD_2018_	DD	DD	DD
Rhinopristiformes	Rhinobatidae	*Rhinobatos holcorhynchus*	Slender Guitarfish		DD_2008_	DD_2018_	DD	DD	DD
Rajiformes	Rajidae	*Dipturus campbelli*	Blackspot Skate		NT_2004_	NT_2019_	LC	LC	NT
Rajiformes	Rajidae	*Dipturus crosnieri*	Madagascar Skate		VU_2006_	VU_2018_	LC	VU	VU
Rajiformes	Rajidae	*Dipturus lanceorostratus*	Rattail Skate		DD_2004_	DD_2018_	DD	DD	DD
Rajiformes	Rajidae	*Dipturus pullopunctatus*	Slime Skate		LC_2004_	LC_2019_	LC	LC	LC
Rajiformes	Rajidae	*Dipturus stenorhynchus*	Prownose Skate		DD_2004_	DD_2018_	DD	DD	DD
Rajiformes	Rajidae	*Leucoraja compagnoi*	Tigertail Skate		DD_2004_	DD_2018_	DD	DD	DD
Rajiformes	Rajidae	*Leucoraja wallacei*	Yellowspotted Skate		LC_2008_	VU_2019_	LC	LC	VU
Rajiformes	Rajidae	*Neoraja stehmanni*	South African Dwarf Skate		DD_2004_	LC_2018_	LC	LC	LC
Rajiformes	Rajidae	*Okamejei heemstrai*	Narrow Skate		DD_2004_	LC_2018_	LC	LC	LC
Rajiformes	Rajidae	*Raja ocellifera*	Twineye Skate			EN_2019_	LC	VU	EN
Rajiformes	Rajidae	*Rajella caudaspinosa*	Munchkin Skate		NT_2004_	LC_2018_	LC	LC	LC
Rajiformes	Rajidae	*Rajella paucispinosa*	Sparsethorn Skate			LC_2018_	LC	LC	LC
Rajiformes	Arhynchobatidae	*Bathyraja smithii*	Softnose Skate		DD_2008_	LC_2019_	LC	LC	LC
Rajiformes	Gurgesiellidae	*Cruriraja durbanensis*	Smoothnose Pygmy Skate		DD_2008_	DD_2018_	DD	DD	DD
Rajiformes	Gurgesiellidae	*Cruriraja hulleyi*	Hulley’s Pygmy Skate		LC_2007_	LC_2018_	LC	LC	LC
Rajiformes	Gurgesiellidae	*Cruriraja parcomaculata*	Roughnose Pygmy Skate		DD_2007_	LC_2018_	LC	LC	LC
Rajiformes	Gurgesiellidae	*Fenestraja maceachrani*	Madagascar Pygmy Skate		DD_2008_	DD_2018_	DD	DD	DD
Rajiformes	Anacanthobatidae	*Anacanthobatis marmorata*	Spotted Legskate		DD_2004_	NT_2019_	LC	LC	NT
Rajiformes	Anacanthobatidae	*Indobatis ori*	Black Legskate		DD_2004_	LC_2019_	LC	LC	LC
Myliobatiformes	Gymnuridae	*Gymnura natalensis*	Diamond Ray		DD_2006_	LC_2018_	LC	LC	LC

(CR, Critically Endangered; EN, Endangered; VU, Vulnerable; NT, Near Threatened; LC, Least Concern; DD, Data Deficient). Species marked with * have been recently described for which previously published assessments do not exist.

### Taxonomic patterns in extinction risk

Nearly one-fifth (*n* = 13, 19%) of assessed endemic sharks and rays in the region are threatened with extinction (Table 1). One species, the Shorttail Nurse Shark (*Pseudoginglymostoma brevicaudatum*), is CR and at an *extremely high* risk of extinction. It is assessed under Criterion A2cd as it has undergone a suspected population reduction of >80% over the past three generation lengths (3GL = 30 years) due to a decline in habitat quality and actual and potential levels of exploitation. Five species (7%) are EN and face a *very high* risk of extinction, and seven species (10%) are VU, facing a *high* risk of extinction (Table 1). A further seven species (10%) are Near Threatened (NT), indicating they may become threatened soon if countries fail to implement fisheries management and conservation measures.

**Fig 2:**
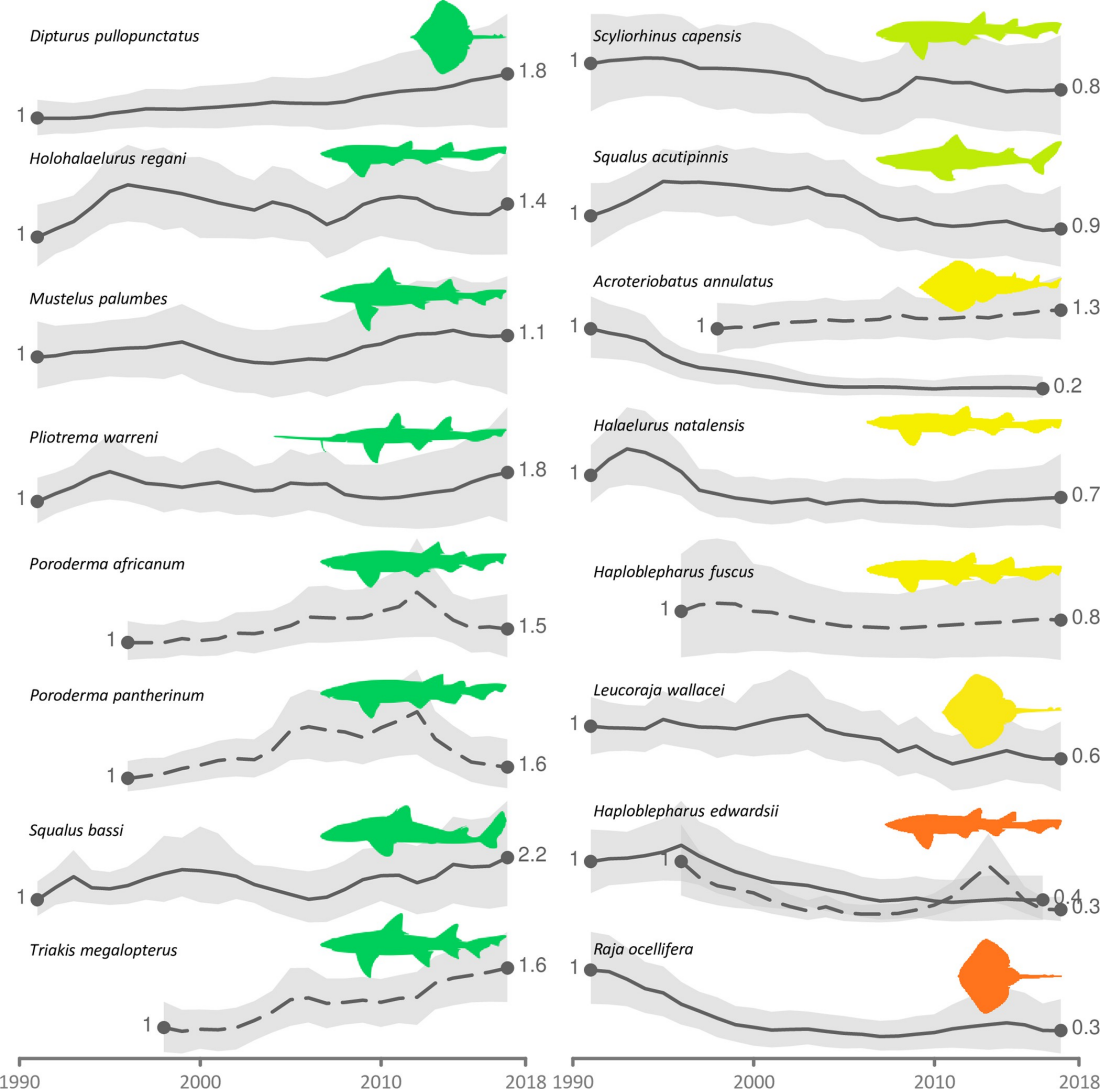
Species population time-series (expressed as a proportion) modelled from demersal research trawl surveys in commercially fished areas (line) and shore-based research angling surveys (dashed line) off the west and south coasts of South Africa. Time-series are normalised so the initial value is 1. Lines and dashed lines denote the mean, and shaded regions represent the 95% credible intervals. Time-series are divided by their initial values and start at one. Silhouette colours indicate Red List status: dark green is Least Concern, light green is Near Threatened, yellow is Vulnerable, orange is Endangered.

Most threatened and NT species were assessed as such using Criterion A (population reduction). For example, the Tiger Catshark (*Halaelurus natalensis*) declined by 39% in the commercial trawl fishing grounds off South Africa in the last 27 years up to 2017, consistent with a population reduction of 56.5% (CI: -97.3, 83.3) over 3GL (60 years, Table 2, and Fig 2). However, there has been an expected range shift away from the trawl grounds reducing catchability in surveys of this area, and experts agreed that the appropriate category for this species is VU. The Lesser Guitarfish (*Acroteriobatus annulatus*) recreational angling CPUE increased by 30% in The De Hoop Marine Protected Area (MPA, dashed line), which was established in 1985 as a no-take reserve. This time-series represents is a smaller inshore fraction of the geographic range of this species and this in part may reflect protection and an inshore shift due to climate change (Fig 4a). By comparison the larger extent of the more representative trawl survey exhibited an 80% decline in CPUE (solid line; Fig 2). This CPUE decline is consistent with a population reduction of 30–49% over the past three generation lengths (15 years), based on the population reduction identified with the trawl survey data, combined with a suspected range shift due to climate change, and therefore was assessed as VU (Table 2). The Twin-eye Skate (*Raja ocellifera*) declined by 65.5% in trawl surveys over the 27 years (1991–2017), consistent with a population reduction of 65.5% (CI: -89.2, -17.1) in 3GL (27 years) and was assessed as EN (Fig 2 and Table 2).

**Table 2:** Endemic SWIO+ shark and ray species and their observed population trend in fisheries trawl surveys and shore-based research angling surveys off the west and south coasts of South Africa and population reduction estimated over three generation lengths (3GL) using JARA (see methods) has been used as a decision-support tool to undertake extinction risk assessments based on the IUCN Red List Categories and Criteria. Table is ordered alphabetically on the Latin binomial.

Species	Common Name	Red List	Survey type	Years	GL	Population trend (%)	
		Category				Observed	3GL
*Acroteriobatus annulatus*	Lesser Guitarfish	VU	trawl	1991–2017	5	-87	-34.1 (-76.7, 62.7)
			angling	1998–2017	5	26	16.7 (-26.3, 75.6)
*Dipturus pullopunctatus*	Slime Skate	LC	trawl	1991–2017	11.5	71	110.1 (10.2, 288.6)
*Halaelurus natalensis*	Tiger Catshark	VU	trawl	1991–2017	20	-39	-56.5 (-97.3, 83.3)
*Haploblepharus edwardsii*	Happy Eddie	EN	trawl	1991–2017	20	-59	-74.8 (-98.9, 36.2)
			angling	1996–2017	20	-72	-92.3 (-99.6, -60.0)
*Haploblepharus fuscus*	Brown Shyshark	VU	angling	1996–2017	20	-21	32.4 (-99.3, 550.6)
*Holohalaelurus regani*	Izak Catshark	LC	trawl	1991–2017	20	39	78.4 (-42.6, 199.4)
*Leucoraja wallacei*	Yellowspotted Skate	VU	trawl	1991–2017	12	-37	-40.4 (-78.6, 32.4)
*Mustelus palumbes*	Whitespotted Smoothhound		trawl	1991–2017	14	15	26.7 (-36.8, 108.4)
*Pliotrema warreni*	Warren’s Sixgill Sawhark	LC	trawl	1991–2017	11	84	96.3 (-55.3, 405.4)
*Poroderma africanum*	Pyjama Catshark	LC	angling	1996–2017	25	30	172.8 (-91.4, 702.0)
*Poroderma pantherinum*	Leopard Catshark	LC	angling	1996–2017	18	64	332.6 (-87.4, 1425.1)
*Raja ocellifera*	Twineye Skate	EN	trawl	1991–2017	9	-70	-65.5 (-89.2, -17.1)
*Scyliorhinus capensis*	Yellowspotted Catshark	NT	trawl	1991–2017	21	-28	-36.3 (-93.4, 115.5)
*Squalus acutipinnis*	Bluntnose Spurdog	NT	trawl	1991–2017	23.5	-12	-21.3 (-78, 95.5)
*Squalus bassi*	African Longnose Spurdog	LC	trawl	1991–2017	23.5	124	146.3 (-33.7, 370.7)
*Triakis megalopterus*	Spotted Gully Shark	LC	angling	1996–2017	20	64	117.7 (55.8, 195.7)

There were two cases of small-range species endemic only to South Africa, found in few locations and undergoing a continuing decline, which were assessed as VU under criterion B: the Natal Shyshark (*Haploblepharus kistnasamyi*) and the Flapnose Houndshark (*Scylliogaleus quecketti*).

Nearly half of species (*n* = 33, 47%) exhibited slight increases or did not decrease substantially enough to meet the criteria for NT or threatened Categories and were thus assigned a status of LC (Table 2). For example, the Softnose Skate (*Bathyraja smithii*) occurs primarily in Namibia, where fishing pressure is relatively low. The Whitecheek Lanternshark (*Etmopterus alphus*) has depth refuge in the absence of deepwater fishing activities as it is found at depths of 472–792 m along a narrow strip of the Mozambique coastline. There were eight species for which indices of abundance were available and revealed either stability or an increase in population index (Fig 2, left column) and these were assessed as LC.

Finally, almost a quarter of species are DD (*n* = 17, 24%) because there is insufficient information to accurately assess their extinction risk (i.e., data are so sparse for these species that assessors were not able to determine whether they are CR or LC, or somewhere between). Three of the six species of guitarfishes from the family Rhinobatidae require further information to assign an extinction risk category. One of five endemic scyliorhinid catsharks and three of 15 pentanchid catsharks are DD. There are fewer data available regarding the status of rays overall, and nearly one-third are DD (10 of 32 species). Three species previously assessed as DD are now LC due to new information: the Saldanha Catshark (*Apristurus saldanha*), the Black Legskate (*Indobatis ori*), and the Whitespotted Smoothhound (*Mustelus palumbes*).

All changes in Red List status since the previous assessments are *non-genuine changes* except for the Shorttail Nurse Shark (which had its population further reduced since the 2005 assessment). These non-genuine changes are due to new information becoming available since the previous assessments. This new knowledge can be used to retrospectively correct previously published assessments for the development of a Red List Index. Thus, the newly stated retrospective statuses can be considered more accurate than the previously published assessments (Table 1).

### Overfishing is identified as the main threat

**Fig 3:**
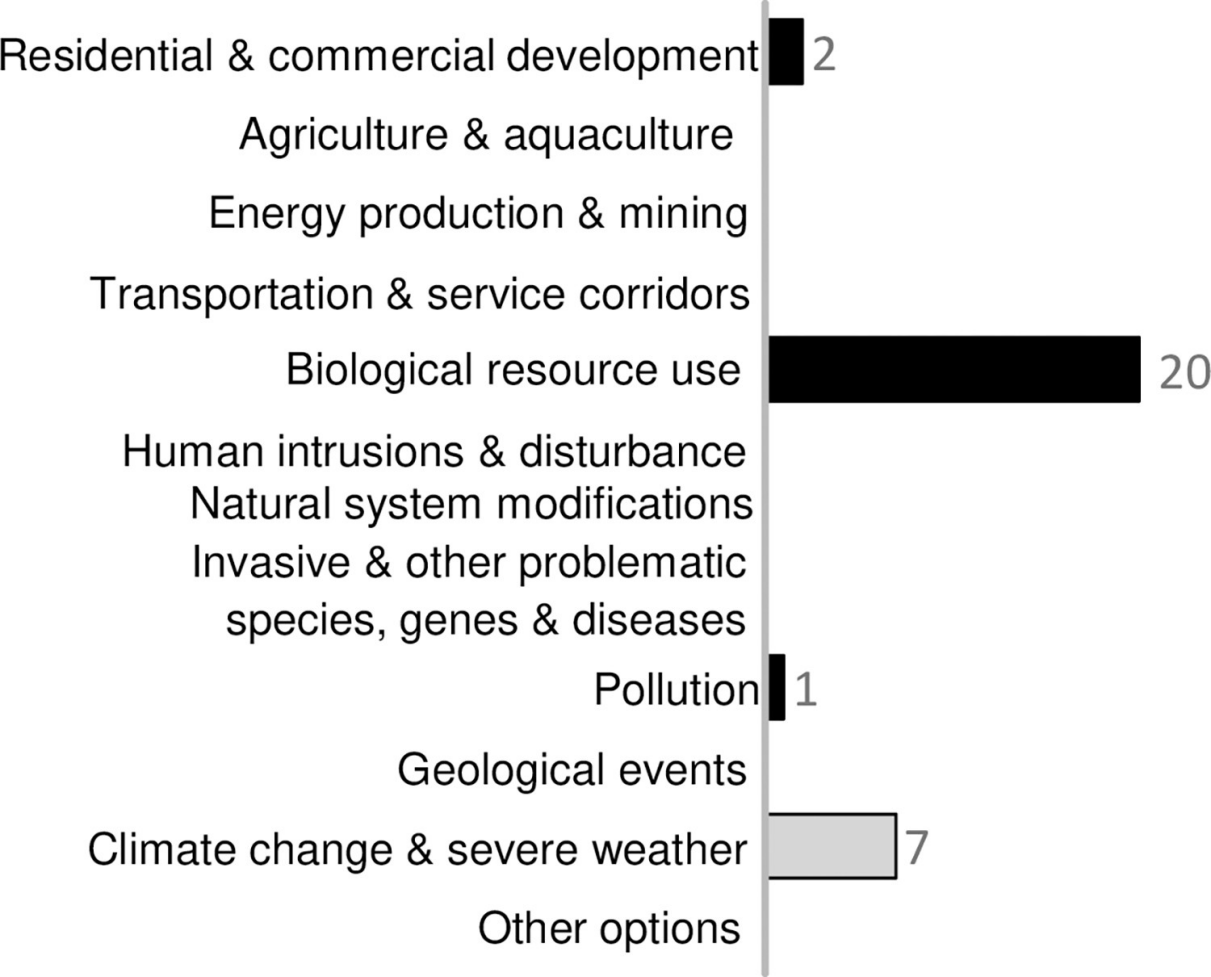
Count of reported threat categories in the 20 threatened (Critically Endangered, Endangered and Vulnerable) and Near Threatened SWIO+ shark and ray species.

Overfishing is identified as the primary threat to all threatened endemic sharks and rays in the SWIO+ region through targeted and incidental catches (bycatch), including commercial, recreational, and artisanal fisheries using fishing gears such as gillnets, longlines, handlines, trawls, and seine nets (Fig 3). Furthermore, all 20 threatened and NT species are exposed to overfishing through incidental catches, where fisheries target other more productive species such as teleost fishes or shrimps but catch and retain other valuable species such as sharks and rays. The identification of overfishing as the primary threat is based on the low prevalence of other threats, and the widespread occurrence of fishing, and capture of species in fisheries which is documented in peer-reviewed articles and grey literature (such as government reports).

Red List assessments for all 13 threatened and seven NT species reported *Biological resource use* and, more specifically, *Fishing & harvesting aquatic resources* as threatening processes (i.e., classified as 5. *Biological resource use*, 5.4 *Fishing & harvesting aquatic resources*), while other threats (e.g., habitat loss and degradation, due to *Residential & commercial development*) caused reductions or a continuing decline in population size in fewer species (Fig 3). *Climate change & severe weather* is reported in the threat rationale for seven threatened and NT species. Although not the leading cause of population reductions, it has induced a significant distributional shift in the populations of six of these seven species [42] (grey bar, Fig 3). Coastal *Residential & commercial development* and *Pollution* contributed to localized extinction risk for three species, but overfishing remains the primary threat for all of them.

### Overfishing is compounded by climate change

**Fig 4:**
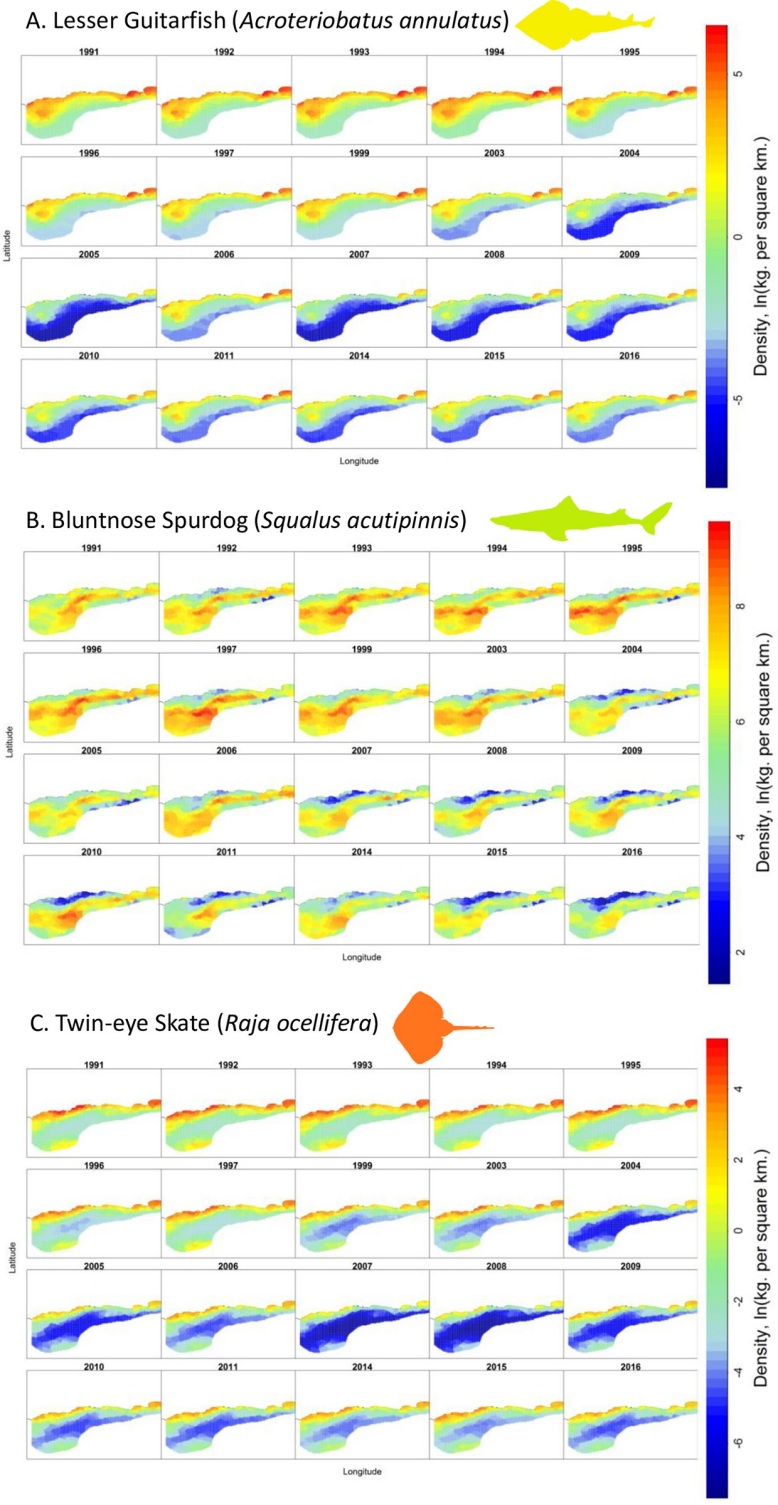
Spatial and temporal change in density (ln kg per km-2) across the South African Shelf from 20°E (Cape Agulhas) and 27°E (Port Alfred) for 1991 and 2016 for (a) Lesser Guitarfish (*Acroteriobatus annulatus*; Vulnerable), (b) Bluntnose Spurdog (*Squalus acutipinnis*; Near Threatened), and (c) Twin-eye Skate (*Raja ocellifera*; Endangered).

There are two impact pathways by which climate change may be elevating extinction risk of sharks and rays in this region. Firstly, the increasing frequency and severity of coral bleaching are implicated in the elevated extinction risk of the Shorttail Nurse Shark. This tropical shark has declined significantly over the past 15 years, resulting in a *genuine* change in status from VU to CR. This population reduction is suspected to be caused by a combination of overfishing, destructive fishing practices, and a continuing decline in habitat quality due to coral bleaching and rising sea temperatures. Secondly, there has been a north-easterly shift in the distribution of thermal habitat across the southern Cape of South Africa. This shift has resulted in simultaneous northeastward shifts in the distributions of many teleost and shark and ray species toward the narrower shelf area off the Eastern Cape and KwaZulu-Natal, along South Africa’s east coast [42]. Three species undergoing notable range shifts are the Lesser Guitarfish VU, Bluntnose Spurdog (*Squalus acutipinnis*) NT, and Twin-eye Skate (*Raja ocellifera*) EN (Fig 4A–4C).

In addition to fishing pressure and climate change, habitat degradation from coastal residential and commercial development and pollution further exacerbates overfishing for two South African endemic catsharks. The Brown Shyshark (*Haploblepharus fuscus*) and the Natal Shyshark both inhabit nearshore waters at depths of less than 50 m. They are endemic to South Africa and occur near several large urban centers (Port Elizabeth, East London, and Durban) and are thus subject to urban development and pollution.

### The Red List Index and national conservation responsibilities

**Fig 5:**
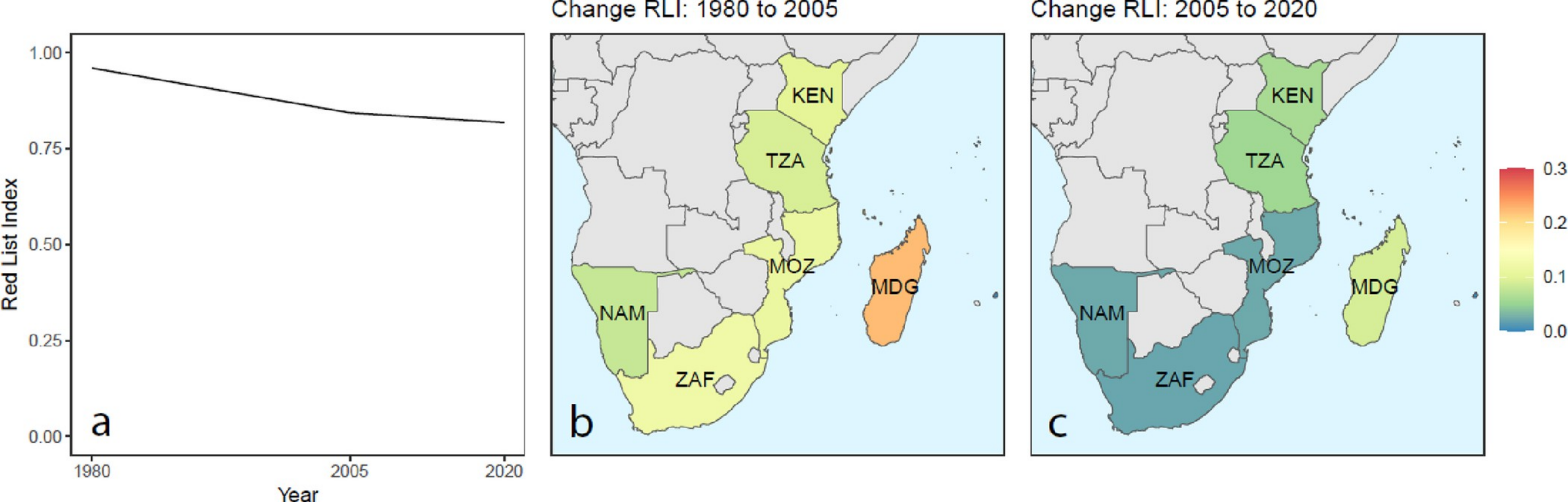
Red List Index (RLI) for SWIO+ endemic sharks and rays (*n* = 70). (a) The decline in RLI across assessment years 1980, 2005, and 2020. Country-specific declines in RLI from (b) 1980–2005 and (c) 2005–2020. Calculations of RLI exclude Data Deficient (DD) species. Maps made with Natural Earth.

Almost all species were retrospectively assessed as LC (*n* = 52) or DD (*n* = 17) in 1980 (Table 1), except one NT species (Natal Sleeper Ray *Heteronarce garmani*), resulting in a regional Red List Index (RLI) value of 0.996 (where a value of 1 represents all assessed species being LC; Fig 5A). The regional RLI decreased slightly to 0.917 by 2005 and further to 0.849 in the most recent assessment (2020) presented here (Fig 5A). This decreasing trend in RLI results from the increased numbers of species in threatened and NT categories by 2005 and 2020 (13 and 20, respectively; Table 1). When disaggregating the RLI down to country-level, the most significant decline in RLI is from 1980 to 2005 in Madagascar (a decline from 0.999 to 0.672; Fig 5B). Between 2005 and 2020, the greatest decline in country-level RLI also occurred in Madagascar (0.672 to 0.558; Fig 5C).

**Fig 6:**
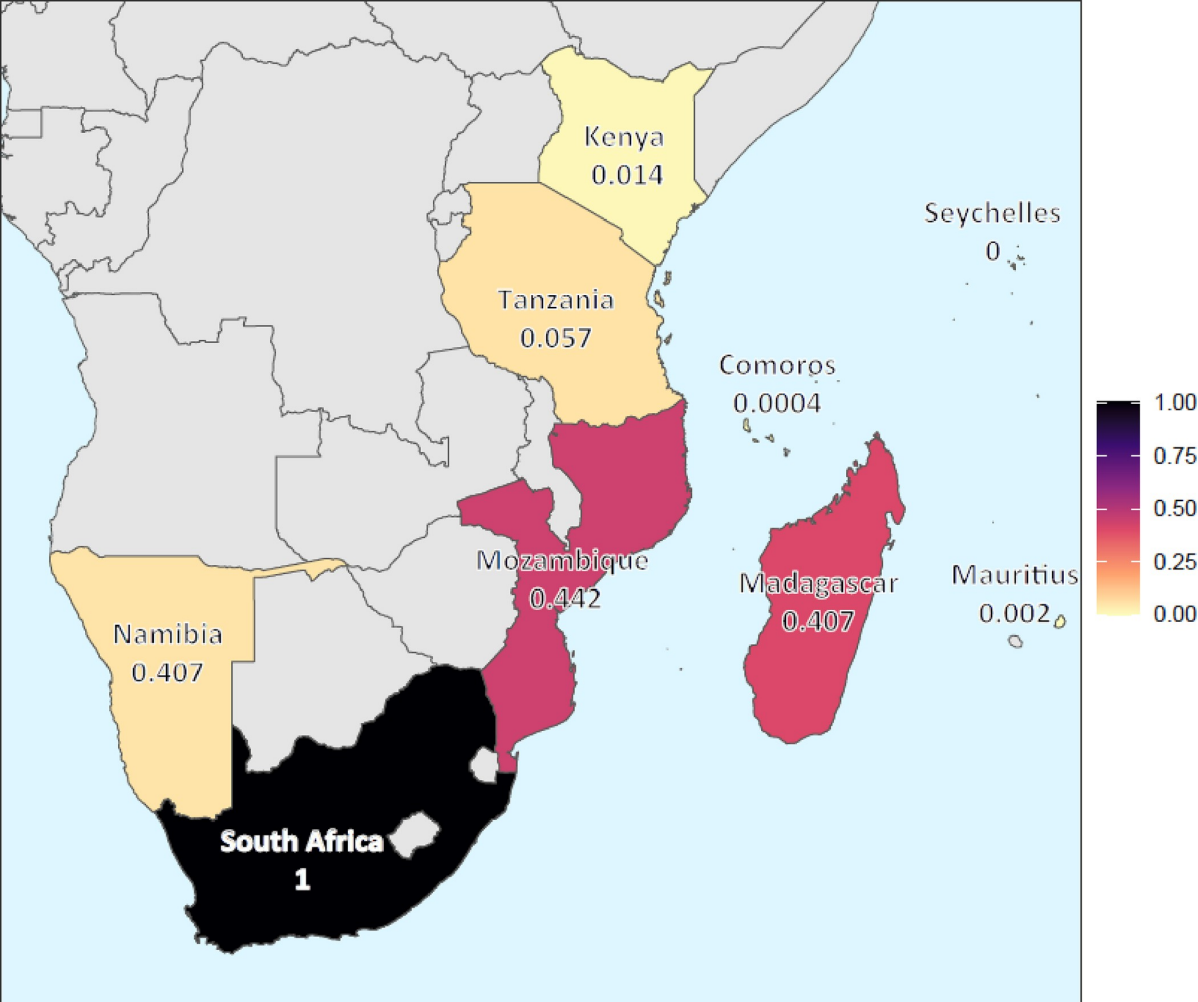
National conservation responsibility of nine range countries for all 70 endemic shark and ray species in the SWIO+ region for which Red List Status is known. Maps made with Natural Earth.

All nine range countries bear some responsibility for conserving the 70 endemic SWIO+ species that have been assessed using the IUCN Red List Categories and Criteria (Fig 6, Table 3). Consistent with the inverse latitudinal trend to greater endemic richness shown in Fig 1, South Africa has the highest national conservation responsibility (NCR) of all nine range countries (NCR = 1), followed by relatively high responsibilities for Mozambique (NCR = 0.442) and Madagascar (NCR = 0.407; Fig 6). Collectively, these three countries represent 93% of all conservation responsibility for endemic sharks and rays in the region, largely reflective of the species richness found in their waters.

**Table 3:** National conservation responsibility of nine range countries for all 70 endemic shark and ray species in the SWIO+region, *excluding Data Deficient species*. Responsibility for each country is calculated based on the numbers of species occurring in the country’s Exclusive Economic Zone (EEZ), the most recent Red List assessment category, and the proportion of each species’ range area occurring in the EEZ (values were normalized to range from 0 to 1).

Country	National Conservation Responsibility
South Africa	1.000
Mozambique	0.442
Madagascar	0.407
Tanzania	0.057
Namibia	0.053
Kenya	0.014
Mauritius	0.002
Comoros	0.001
Seychelles	0.000

## Discussion

Here, we provide the first comprehensive reassessment of extinction risk in sharks and rays that are endemic to waters of the SWIO+ region. Of 70 species herein assessed for the IUCN Red List, nearly one-fifth are threatened and thus have a high to extremely high risk of extinction (1 CR, 5 EN, 7 VU). Despite a lack of data from parts of the region, excessive fishing activity and limited management capacity are substantial barriers to ensuring robust shark and ray populations into the future. A further quarter of species are DD and could potentially be listed as threatened as additional data become available. Furthermore, this assessment of endemic species belies the overall status of all sharks and rays in the region because it does not include wider-ranging or highly-mobile species that may be fished even more heavily here or face additional fishing pressure from other regions [59]. We next (1) compare these findings to threat patterns globally and in other regions and identify measures to: (2) avoid extinctions, (3) ensure sustainability, (4) maintain robust functional populations, (5) drive down data deficiency, and (6) cope with prevalent and emerging threats.

### Comparison of SWIO+ endemic threat to other regions

The percentage of threatened endemic species in this region (19%) is considerably lower than that estimated globally (37.5%) [4]. At the regional level (including all species wide-ranging and endemic), 42% of all species (*n* = 50) are threatened or predicted to be threatened in the Northwest Atlantic and two-thirds of all species (67%, *n* = 48) in the Mediterranean Sea [14]. A regional assessment (including all species wide-ranging and endemic) of the Arabian Sea and its adjacent waters found 50.9% of species are threatened [29]. Although we find extinction risk of endemic sharks and rays in SWIO+ to be lower than in these other regions, many of the most threatened families found in this region are not included in this assessment because they are not endemics, including sawfishes, wedgefishes, hammerheads, and thresher sharks [13, 15, 60]. If they are included, there are 85 of 240 species (35.4%) threatened in this SWIO+ region based on the IUCN Red List 2022–2 [5]. Nevertheless, endemicity adds a further layer of extinction risk to a species and we show here that there are at least six endemic species that are in the highest threat categories (EN or CR) and require urgent conservation action to prevent further declines and extinction.

### Avoiding extinctions

The most severe and prevalent threat to the endemic species assessed in this region is heavy fishing pressure and bycatch mortality, resulting in population reductions for threatened and NT species. This threat is particularly problematic for species inhabiting shallow inshore and continental shelf waters to approximately 200 m depth, such as the Shorttail Nurse Shark (the only CR species), and the Happy Eddie Catshark (*Haploblepharus edwardsii)*, Greyspot Guitarfish (*Acroteriobatus leucospilus*), and Twin-eye Skate (all EN). In the specific case of the Shorttail Nurse Shark, extensive landings surveys in Madagascar (2007–2012) have not recorded any individuals [61], and only one individual has been observed there in 270 hours of baited remote underwater video (BRUV) surveys (Wildlife Conservation Society. Unpublished Data, 2018), although since this assessment, its range has been extended to include Mozambique [62] and South Africa (Bennett, R. unpubl. data). Further, no sightings of this species have been reported from catch surveys of a wide range of artisanal fishing gears in Kenya, Zanzibar, and northern Madagascar (2016–2017) [26, 27], as well as extensive visual census surveys in Tanzania, Mozambique, or Madagascar (2009–2015) [63]. However, observations since this assessment suggest that this species is being targeted in Kenya for export for the aquarium trade, although the numbers of individuals captured and traded remain unknown (Bennett, R. unpubl. data).

We recommend that governments implement management interventions for CR and EN species without delay. Priority interventions would include strict prohibitions on landings, where they are not yet in place, along with appropriate capacity-building for communication and enforcement. Priority effort could be focused on understanding and reducing catch in those gears that cause greatest mortality, such as large-mesh (shark-directed) gillnets and longlines. Regulation of destructive fishing practices such as the use of reef nets and blast fishing, which damage habitats such as coral reefs, should be implemented and enforced. If threats are not mitigated rapidly, species such as the Shorttail Nurse Shark could become extinct in the very near future. This situation could follow that of at least one, possibly two, sawfish species that are already considered locally extinct in South Africa (Largetooth Sawfish *Pristis pristis* and Green Sawfish *P*. *zijsron*) [64]. Although they are the first rays protected in the region, protection was implemented too late, two years before the last sighting of a sawfish in South Africa [64].

### Ensuring sustainability

Marine Protected Areas might prove to be a suitable approach for conserving threatened endemic sharks, such as the Flapnose Houndshark and Natal Shyshark, which have small geographic range sizes, occur in few locations, and are inferred to have declining populations [65, 66]. Establishing closures will require the identification of overlap between the existing protected area network and key habitat features and understanding movement behaviour and potential aggregation sites [67–69]. Even a modest expansion of the protected areas network has significant potential to contribute to the conservation of these species [70, 71]. The implementation of spatial closures of important habitat could complement catch and fishing effort reduction approaches.

Madagascar, South Africa, and Seychelles are the only nations in the region with a National Plan of Action (NPOA) for the Conservation and Management of Sharks [72, 73], although most SWIO+ countries are in the process of developing these. From our analyses, Mozambique and Madagascar had the most significant national conservation responsibility after South Africa, with these three nations representing 93% of all responsibility in the region. Given its high national conservation priority, we recommend that Mozambique undertakes the necessary steps to finalise the development of, and implement, its NPOA. Further, such plans should include legislative mechanisms for protection of CR and EN species, explicit actions on catch limits for VU or NT species, strategies for managing bycatch in fisheries and, where needed, actions on protecting habitats or areas known as important during critical life history stages [74]. Increased efforts to accurately assess fishing pressure are also paramount. Underreporting and discrepancies in fisheries data are prevalent in reports provided to Regional Fisheries Management Organisations (RFMOs) and the Food and Agriculture Organization of the United Nations (FAO) [75–77]. Furthermore, where data are collected, discards are not reported, and post-release mortality is poorly understood, even in South Africa, where data collection is relatively robust [72].

### Maintaining robust and ecologically functional populations

Encouragingly, almost half of the species assessed here are LC, which means their populations are stable or declining slowly such that population reduction thresholds are not triggered. In many cases, these species are not exposed to the pressures to which threatened species are. For example, the geographic or bathymetric ranges of some species mean they are sparsely or never fished. Where a species is fished, resilience to this pressure is indicated by relatively stable population trends over time. For example, the Whitespot Smoothhound (*Mustelus palumbes*) has shown a modest estimated increase of 8% over 27 years across the South African commercial trawl grounds (Fig 2). Whitespotted Smoothhound is caught in trawl, line, and demersal shark longline fisheries, but given its increase in abundance, the species appears to be resilient to moderate levels of fishing activity (< 50 t per annum), although further management measures will be needed to ensure sustainability if catches increase. Some other targeted or retained bycatch species (e.g., Bluntnose Spurdog *Squalus acutipinnis*, Slime Skate *Dipturus pullopunctatus*) also exhibit some level of resilience to fishing pressure. However, it is essential that these LC species be monitored in terms of abundance and catch to maintain robust, ecologically functional populations that yield ecosystem services to humanity and contribute to food security.

### Driving down data deficiency

A quarter of the species assessed had insufficient data available for an accurate assessment and were evaluated as DD. The region generally has among the highest levels of shark and ray data deficiency globally [4]. Many countries are still reporting catches simply as ‘sharks’, and species-level monitoring of rays has been particularly neglected in the region. Catch reconstructions reveal serious discrepancies where reported catches are far lower than the reconstructions, around 200% in Madagascar and Mauritius, and >75% in Tanzania [74, 78, 79]. While there has been progress in assessing the species composition and monitoring of fisheries, there remains a lack of species-specific population trend and time-series data, other than in South Africa. The lack of species-specific fisheries data means that declines in sensitive species (e.g., angelsharks, guitarfishes) could go unnoticed [80, 81]. More information may reveal other species that are threatened. More detailed, species-specific information is needed to provide effective spatial planning and fisheries management while minimizing impacts and conflicts with resource users.

### Coping with emerging threats

Emerging threats in this region include the expansion of deepwater fisheries and climate change impacts on sharks and rays. Two deepwater species affected by fishing pressure are EN catsharks: the Honeycomb Izak Catshark (*Holohalaelurus favus*) and the African Spotted Catshark (*H*. *punctatus*), which occur in waters greater than 200 m. Despite the potential for refuge at depth, populations of these deepwater catsharks are suspected of having undergone reductions of more than 50% over the past 3GL due to deepwater trawl and longline fisheries operating within their ranges. These declines will continue if deepwater fisheries are further developed in the absence of management for both target and bycatch species [82]. We caution that as deepwater fisheries increase, particularly in Mozambique, Madagascar, and Tanzania, including fishing by distant water nations, many of the deepwater LC species may be at greater risk of extinction. Monitoring fisheries expansions into deeper or more remote waters overlapping with the geographic ranges of deepwater LC species will be important, along with the recording and reporting of species-level catch data.

Although declines in VU species are mainly due to fishing, several species are likely to have undergone a population reduction (including a reduction in area of occupancy) that is at least partially related to an ecological shift in ocean currents due to climate change [42, 83]. For example, for the Tiger Catshark, mortality due to fisheries does not appear substantial enough to be the only factor causing this reduction, highlighting the importance of considering climate change in future Red List assessments of sharks and rays [84]. Another example is the Lesser Guitarfish, for which the estimated reduction is driven partly by a steep decline in catch rates during the early 1990s when fishing pressure in South Africa was substantially higher; over the last two decades, the population reduction has been less dramatic. Some of the recent reduction is likely a result of a climate-driven north-east range shift of the species away from the core offshore trawl survey area into less-surveyed inshore habitats (Fig 4A). This range shift also likely represents a significant range contraction of the Lesser Guitarfish and a reduction in area of occupancy. The De Hoop MPA, for which data indicate a slight increasing trend that conflicts with the trawl data for this species, was established in 1985 and is a no-take reserve; while this may not be representative of the population trends in fished areas of South Africa, the population increase there may reflect an inshore range shift by the Lesser Guitarfish in response to climate change.

As species distribution models for sharks and rays become available [71], future assessments could consider using climate projections. Trait-based approaches are already available to evaluate the potential risk of climate change and will be helpful for future reassessment [84].

## Conclusion

Here, we find that one-fifth (13, 19%) of the 70 endemic shark and ray species in the SWIO+ region are threatened with extinction. There is thus a need for a collaborative regional improvement in shark and ray conservation to reduce risk for these endemic species. The uniqueness of this SWIO+ endemic hotspot for sharks and rays is of global significance and requires international support. There is a great urgency to act to avoid extinctions, ensure sustainability, maintain robust functional populations, reduce data deficiency, and thereby help to secure livelihoods and food security for coastal people. Long-term monitoring and data collection at the species level are essential, particularly for threatened and NT species. Species-specific annual fisheries-independent population monitoring needs to take place. In the absence of such data, species-specific monitoring of catches and landings (accounting for fishing effort) can provide a reliable index of the trend in abundance. Although there has been an improvement in fisheries management in South Africa, and it may well have maintained populations and prevented more severe declines than those observed, many other countries in the region lag far behind. These nations need to significantly improve their capacity to effectively monitor, manage, and protect their shark and ray species and play their part to ensure the global viability of shark and ray fauna. The new Kunming-Montreal Global Biodiversity Framework has the goal of halting and reversing declines in populations and minimizing extinctions [6, 85]. This study provides the first evidence that marine extinction risk has increased in the SWIO+ region due to overfishing and climate change and that action is needed to safeguard the future of these iconic endemic sharks and rays.
